# Serum Proteomics and Plasma Fibulin-3 in Differentiation of Mesothelioma From Asbestos-Exposed Controls and Patients With Other Pleural Diseases

**DOI:** 10.1016/j.jtho.2021.05.018

**Published:** 2021-10

**Authors:** Selina Tsim, Laura Alexander, Caroline Kelly, Ann Shaw, Samantha Hinsley, Stephen Clark, Matthew Evison, Jayne Holme, Euan J. Cameron, Davand Sharma, Angela Wright, Seamus Grundy, Douglas Grieve, Alina Ionescu, David P. Breen, Elankumaran Paramasivam, Ioannis Psallidas, Dipak Mukherjee, Mahendran Chetty, Giles Cox, Alan Hart-Thomas, Rehan Naseer, John Edwards, Cyrus Daneshvar, Rakesh Panchal, Mohammed Munavvar, Rachel Ostroff, Leigh Alexander, Holly Hall, Matthew Neilson, Crispin Miller, Carol McCormick, Fiona Thomson, Anthony J. Chalmers, Nick A. Maskell, Kevin G. Blyth

**Affiliations:** a Glasgow Pleural Disease Unit, Queen Elizabeth University Hospital, Glasgow, United Kingdom; b Institute of Cancer Sciences, University of Glasgow, Glasgow, United Kingdom; c Cancer Research UK Clinical Trials Unit Glasgow, University of Glasgow, Glasgow, United Kingdom; d Department of Respiratory Medicine, University Hospital of South Manchester, United Kingdom; e Department of Respiratory Medicine, Forth Valley Royal Hospital, Larbert, United Kingdom; f Department of Respiratory Medicine, Inverclyde Royal Hospital, Greenock, United Kingdom; g Department of Respiratory Medicine, Glasgow Royal Infirmary, Glasgow, United Kingdom; h Department of Respiratory Medicine, Salford Royal Hospital, Salford, United Kingdom; i Department of Respiratory Medicine, Royal Alexandra Hospital, Paisley, United Kingdom; j Department of Respiratory Medicine, Royal Gwent Hospital, Newport, United Kingdom; k Department of Respiratory Medicine, Galway University Hospital, Galway, Ireland; l Department of Respiratory Medicine, St James’s University Hospital, Leeds, United Kingdom; m Oxford Centre for Respiratory Medicine, Churchill Hospital, Oxford, United Kingdom; n Department of Respiratory Medicine, Basildon University Hospital, Basildon, United Kingdom; o Department of Respiratory Medicine, Aberdeen Royal Infirmary, Aberdeen, United Kingdom; p Department of Respiratory Medicine, King’s Mill Hospital, Sutton-in-Ashfield, United Kingdom; q Department of Respiratory Medicine, Huddersfield Royal Infirmary, Huddersfield, United Kingdom; r Department of Cardiothoracic Surgery, Northern General Hospital, Sheffield, United Kingdom; s Department of Respiratory Medicine, Derriford Hospital, Plymouth, United Kingdom; t Department of Respiratory Medicine, Glenfield Hospital, Leicester, United Kingdom; u Department of Respiratory Medicine, Royal Preston Hospital, Preston, United Kingdom; v SomaLogic Inc., Boulder, Colorado; w Cancer Research UK Beatson Institute, Glasgow, United Kingdom; x Academic Respiratory Unit, Bristol Medical School, University of Bristol, Bristol, United Kingdom

**Keywords:** Mesothelioma, SOMAscan, Fibulin-3, Mesothelin, Biomarker

## Abstract

**Introduction:**

Malignant pleural mesothelioma (MPM) is difficult to diagnose. An accurate blood biomarker could prompt specialist referral or be deployed in future screening. In earlier retrospective studies, SOMAscan proteomics (Somalogic, Boulder, CO) and fibulin-3 seemed highly accurate, but SOMAscan has not been validated prospectively and subsequent fibulin-3 data have been contradictory.

**Methods:**

A multicenter prospective observational study was performed in 22 centers, generating a large intention-to-diagnose cohort. Blood sampling, processing, and diagnostic assessment were standardized, including a 1-year follow-up. Plasma fibulin-3 was measured using two enzyme-linked immunosorbent assays (CloudClone [used in previous studies] and BosterBio, Pleasanton, CA). Serum proteomics was measured using the SOMAscan assay. Diagnostic performance (sensitivity at 95% specificity, area under the curve [AUC]) was benchmarked against serum mesothelin (Mesomark, Fujirebio Diagnostics, Malvern, PA). Biomarkers were correlated against primary tumor volume, inflammatory markers, and asbestos exposure.

**Results:**

A total of 638 patients with suspected pleural malignancy (SPM) and 110 asbestos-exposed controls (AECs) were recruited. SOMAscan reliably differentiated MPM from AECs (75% sensitivity, 88.2% specificity, validation cohort AUC 0.855) but was not useful in patients with differentiating non-MPM SPM. Fibulin-3 (by BosterBio after failed CloudClone validation) revealed 7.4% and 11.9% sensitivity at 95% specificity in MPM versus non-MPM SPM and AECs, respectively (associated AUCs 0.611 [0.557–0.664], *p* = 0.0015) and 0.516 [0.443–0.589], *p* = 0.671), both inferior to mesothelin. SOMAscan proteins correlated with inflammatory markers but not with asbestos exposure. Neither biomarker correlated with tumor volume.

**Conclusions:**

SOMAscan may prove useful as a future screening test for MPM in asbestos-exposed persons. Neither fibulin-3 nor SOMAscan should be used for diagnosis or pathway stratification.

## Introduction

Malignant pleural mesothelioma (MPM) is an invasive thoracic malignancy strongly associated with asbestos exposure. The diagnosis of MPM is often difficult because the disease presents nonspecifically with a pleural effusion or mass, and tumors are not easily biopsied in early-stage disease.^1^ An accurate blood biomarker would be a considerable clinical advancement but would require high sensitivity and high specificity given the low incidence of MPM in most settings. In a retrospective study, the secreted glycoprotein fibulin-3 had 96.7% sensitivity at 95.5% specificity for MPM,^2^ but subsequent studies have reported conflicting results,^3^, ^4^, ^5^, ^6^ leaving uncertainty regarding its value.^7^^,^^8^ The SOMAscan proteomic assay (Somalogic, Boulder, CO) was also associated with high (93.2%) sensitivity and specificity (90.8%) in a similar retrospective study,^9^ but has yet to be evaluated prospectively. Here, we report results from the Diagnostic and Prognostic Biomarkers in the Rational Assessment of Mesothelioma (DIAPHRAGM) study, which was a prospective, multicenter observational study designed to validate fibulin-3 and SOMAscan and benchmark performance against mesothelin, which has been widely studied but offers only 32% sensitivity at 95% specificity.^10^ To our knowledge, DIAPHRAGM was the largest prospective, intention-to-diagnose MPM biomarker study ever conducted.

## Materials and Methods

### Study Design

The study protocol was previously published^11^ and was compliant with the Standards for Reporting Diagnostic Accuracy guidelines.^12^ The primary objective was to determine whether SOMAscan or fibulin-3 could differentiate MPM from asbestos-exposed controls (AECs) or other patients with suspected pleural malignancy (SPM) with sufficient sensitivity and specificity to be of routine clinical value. Secondary objectives related to prognostic value will be reported separately. Exploratory objectives regarding associations with primary tumor volume, defined by magnetic resonance imaging (MRI) and a range of potential confounders are reported here (age, sex, renal function [as estimated glomerular filtration rate (eGFR)], previous asbestos exposure, C-reactive protein [CRP], white cell count [WCC], timing of blood draw). The study received ethics committee approval (reference 13/WS/0240) and was registered (ISRCTN100799720).

### Participants

Patients with SPM were recruited from 22 centers in the United Kingdom and Ireland between December 31, 2013 and December 31, 2016. Potential cases were identified on presentation to secondary care. AECs were recruited in Glasgow from respiratory medicine clinics and an asbestos advocacy body.

#### Eligibility Criteria

SPM cases required a new unilateral pleural effusion or mass, sufficient fitness for diagnostic sampling, and informed written consent. Patients with current or recent chest drain (≤3 mo) were ineligible. Participants with SPM in Glasgow were eligible for a substudy addressing the exploratory MRI objectives if they required histologic sampling and did not report MRI contraindications. AECs required a history of asbestos exposure with evidence of pleural plaques, asbestosis, or diffuse pleural thickening. AECs with known or suspected malignancy or pleural effusion were excluded.

#### Diagnostic Assessment and Follow-Up

All patients with SPM underwent robust diagnostic assessment (Supplementary Appendix Fig. A1). All centers had access to local anesthetic thoracoscopy and a specialist mesothelioma multidisciplinary team. The disease stage was recorded using TNM version 7, as was standard practice at study initiation. All patients with a benign diagnosis had a mandatory 12-month diagnostic review, acknowledging the diagnostic difficulties inherent to MPM and the potential for MPM evolution in a minority.^13^ Any patient who developed MPM within 12-months was labeled an “evolver” and excluded from the primary analyses.

### Sample Size

Sample size calculations were described in detail in the published protocol.^11^ These were based on previously published data regarding each marker and a projected MPM incidence in the SPM cohort of 13% to 20%. The target sample size was 600 SPM cases, including at least 83 cases of MPM (13% incidence) and 109 AEC. The power available to test several hypotheses related to sensitivity values (at high specificity) above or below a priori definitions of “clinically useful” was ultimately dependent on the final number of MPM cases included in each assay cohort.^11^ The target sample size for the MRI substudy was 20 patients with MPM, allowing relatively large associations (r ≥ 0.6) between tumor volume and biomarker levels to be reliably detected.^11^

### Test Methods

#### Sampling and Processing

Blood sampling was designed to replicate clinical practice and was performed before pleural biopsies or treatment (Supplementary Appendix Fig. A1). A total of 18 mL of venous blood was collected into vacutainers containing serum separator tubes (SST) clot activator (9 mL) or ethylenediaminetetraacetic acid (EDTA) (9 mL). Blood in SST tubes was allowed to clot for 30 minutes before centrifugation. EDTA tubes were spun immediately. All samples were centrifuged at 2200 g for 15 minutes at room temperature, after which serum (from SST) and plasma (from EDTA) were aliquoted into cryovials. Samples were stored at −80°C within 2 hours of blood draw before batched transport to Glasgow for banking.

#### Selection of Samples for Assay Cohorts

Once all samples had been banked, cryovials were selected for each assay. Although serum and plasma were available for each patient, only one sample type was selected for each assay as per the manufacturers’ instructions. This process was managed to ensure the sample sizes required for each evaluation were available. For fibulin-3 and mesothelin, MPM, and AEC samples were always selected, when available; non-MPM SPM samples were selected at random, aiming for at least the number prespecified in sample size calculations (n = 378),^11^ up to a maximum number determined by available funding. For SOMAscan proteomics, budgetary constraints limited numbers to 120 MPM, 83 non-MPM SPMs, and 83 AECs, which were selected at random. Diagnosis quality control was conducted in parallel, allowing evolvers to be replaced.

#### Biomarker Assays

##### Fibulin-3

Fibulin-3 assays were measured in plasma in accordance with the manufacturers’ instructions. The performance of the USCN CloudClone enzyme-linked immunosorbent assay (ELISA) (Wuhan, People’s Republic of China, distributed by USCN Life Sciences, Houston, TX) used in previous MPM studies^2^, ^3^, ^4^, ^5^, ^6^ was first evaluated according to regulatory authority guideline.^14^ However, assay performance was deemed unacceptable owing to poor intraassay and interassay reproducibility (Supplementary Appendix Fig. A2). An alternative ELISA (BosterBio, Pleasanton, CA) was therefore sourced and evaluated. This assay exhibited acceptable performance and was used throughout (Supplementary Appendix Fig. A2). Each sample was measured in duplicate wells on at least two occasions (i.e., four replicates), generating a mean value and coefficient of variation (CV). Samples with a CV greater than 30% were considered to have an unacceptable degree of variability and excluded from the final analysis. If the measured concentration was greater than the upper limit of quantification for the assay, the sample was diluted and reanalyzed.

##### SOMAscan Proteomics

The SOMAscan proteomic assay utilizes modified DNA aptamers, termed SOMAmers, to bind 1305 proteins within 65 μL of serum, using a bead-based microarray (version 3).^9^^,^^15^ This generates an output in relative fluorescent units, which is directly proportional to the amount of target protein in the initial sample. Ostroff et al.^10^ previously reported a 13-protein MPM signature using assay versions 1 and 2. An update to version 3 during recruitment to the current study included the replacement of one of the original proteins (fibronectin 1, replaced by fibronectin fragment 4). This version was used for all analyses reported. Samples were analyzed by SomaLogic (Boulder, CO), who was blinded to clinical details. Normalization was done by aligning the median of each sample to a common reference. Interplate calibration was done by applying a multiplicative scaling coefficient to each SOMAmer. These scaling factors were calculated using eight reference calibrators on each plate.

##### Mesothelin

Mesothelin measured in serum using the Mesomark ELISA (Fujirebio Diagnostics, Malvern, PA). Assay performance was initially evaluated according to regulatory authority guidelines^14^ before measurements were made, according to the manufacturer’s instructions. The methods used, including the number of replicates and handling of CV values, were as described for fibulin-3. Results below the lower limit of quantification (LLOQ) (2 nM) of the Mesomark assay were common. If individual replicates were less than LLOQ, these were recorded as less than 2 nM. Cases were excluded from statistical analyses if the variability of the four replicates was too great or the proximity of an imprecise mean to the previously validated MPM cut-point (2.5 nM^3,^^16^^,^^17^) made it unclassifiable (e.g., <3).

#### Volumetric MRI

MRI acquisition and volumetric methods have previously been published.^18^^,^^19^ Primary tumor volume was recorded in cm^3^.

### Statistical Analysis

The statistical analysis plan was included in the published protocol.^11^ Data are reported as median (interquartile range) on the basis of distribution unless otherwise stated. Mesothelin values were computed by imputation, values less than LLOQ were replaced by 1.0 nM (half the LLOQ). Disease group comparisons used Kruskal-Wallis tests, with Dunn’s test for subgroup comparisons. Fibulin-3 and mesothelin performance were quantified using receiver operator characteristic (ROC) curves, with sensitivity reported at a prespecified level of 95% specificity. Per protocol, validation of the previous fibulin-3 cut-point (52 ng/mL)^3^ on the basis of the CloudClone ELISA was not possible; therefore, optimum performance was reported on the basis of Youden’s index.^18^ Mesothelin performance was reported at the previously validated 2.5 nM cut-point.^4^^,^^19^^,^^20^ Sensitivity analyses determined the effect on mesothelin ROC and disease group comparisons of three alternative methods of handling less than LLOQ mesothelin values, and (3) the effect on fibulin-3 and mesothelin ROCs when excluded evolver cases were included and classified as either MPM or non-MPM SPM.

Per protocol, validation was not possible owing to the revision of the assay (to version 3) during recruitment. Differential expression of the 1305 SOMAscan proteins was reported using the Limma (version 3.46) R package,^20^^,^^21^ including the 13 proteins in the previous signature (with fibronectin 1 replaced by fibronectin fragment 4), and summarized by volcano plots. Gradient boosted logistic regression models were constructed if univariate analysis seemed discriminatory, with disease groups (MPM versus AECs or MPM versus non-MPM SPM) split 80-to-20 into training and internal validation sets. Five repeats of 10-fold cross-validation were used for training. Model performance was evaluated and reported as AUC (95% DeLong confidence interval) and optimal sensitivity and specificity. Evolver data was included in differential expression, but these cases did not contribute to group classification. Evolvers were excluded from the gradient boosted logistic regression models.

Associations between fibulin-3, SOMAscan constituent proteins, and mesothelin were estimated by Spearman or Mann-Whitney *U* test. Statistically significant associations (at the 5% level) were reported if they remained significant after Benjamini and Hochberg adjustment for multiple comparisons.^21^ Statistical Package for the Social Sciences version 24, Statistical Analysis System version 9.4, R version 3.5.1, and Prism version 8 for Mac were used.

## Results

### Participants

#### Study Population

**Figure 1:**
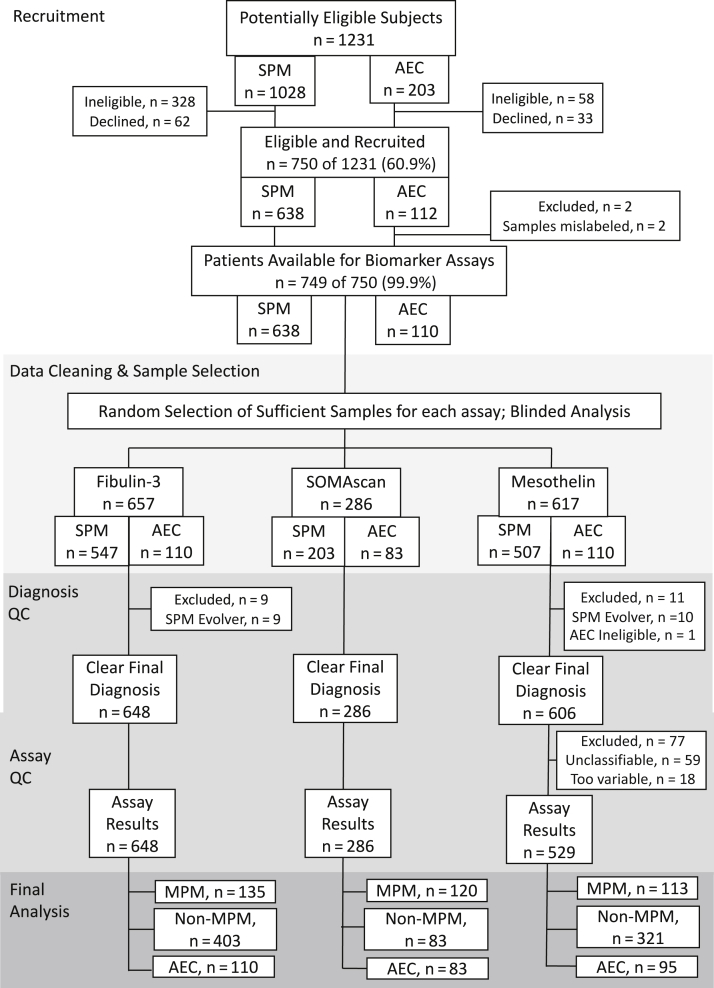
Study flowchart summarizing patient recruitment, sample selection for assay cohorts, diagnosis and assay QC steps including reasons for exclusion from the final analysis, and disease group memberships. AEC, asbestos-exposed control; MPM, Malignant pleural mesothelioma; QC, quality control; SPM, suspected pleural malignancy.

A total of 638 SPM and 112 AEC were recruited (Fig. 1). A total of 16 of 638 SPM cases (2.5%) were classified as evolvers and excluded from the primary analysis. The final diagnoses in the remaining 622 patients were as follows: (1) MPM in 152 of 622 (24.4%); (2) secondary pleural malignancy in 218 of 622 (35.1%); and (3) benign pleural disease in 252 of 622 (40.5%). MPM diagnoses were based on histological features in 129 of 152 (84.9%), radiology and cytology in 10 of 152 (6.6%), radiology in 9 of 152 (5.9%), and postmortem findings in 1 of 152 (0.7%). TNM staging was I in 52 of 152 (34.2%), II in 14 of 152 (9.2%), III in 58 of 152 (38.2%), IV in 23 of 152 (15.1%), and not recorded in 5 of 152 (3.2%).

#### Sample Banking and Selection for Assays

A total of 5884 sample aliquots (2883 serum, 3001 plasma) were banked, allowing the selection of three assay cohorts (Fig. 1). These were well matched for demographics, disease stage (in MPM cases), and other clinical characteristics (Table 1).

**Table 1:** Demographics, Clinical Features With and Without Mesothelioma Staging of each Assay Cohort

Assay Cohort	Fibulin-3	SOMAscan	Mesothelin
Mesothelioma			
Number	n=135	n=119	n=113
Age	75 (68-81)	75 (68-81)	76 (70-81)
Male Sex	120/135 (89%)	105/119 (88%)	100/113 (89%)
Known Asbestos Exposure	112/135 (83%)	96/119 (81%)	92/113 (81%)
Epithelioid Histological sub-type	75/135 (56%)	68/119 (57%)	61/113 (54%)
Disease Stage^a^			
*I*	41/131 (31%)	36/119 (30%)	32/109 (29%)
*II*	14/131 (11%)	14/119 (12%)	10/109 (9%)
*III*	55/131 (42%)	52/119 (44%)	45/109 (41%)
*IV*	21/131 (16%)	17/119 (14%)	22/109 (20%)
eGFR (ml/min)	74 (61-90)	73 (61-90)	73 (61-88)
Sampled preaspiration	56/132 (42%)	48/116 (41%)	48/110 (44%)
WCC (1 x 10^9^/L)	8.3 (6.9-9.8)	8.3 (7.0-9.8)	8.3 (7.2-9.8)
CRP (mg/L)	18 (5-62)	17 (5-62)	17 (5-62)
Non-Mesothelioma SPM^b^			
Number	n=403	n=80	n=321
Age	72 (65-80)	73 (64-79)	72 (65-79)
Male Sex	282/403 (70%)	59/80 (74%)	219/321 (68%)
Known Asbestos Exposure	176/403 (44%)	33/80 (41%)	134/321 (42%)
eGFR (mL/min)	77 (55-100)	76 (55-107)	77 (55-98)
Sampled preaspiration	175/382 (46%)	39/76 (51%)	140/303 (46%)
WCC (1 x 10^9^/L)	9.0 (7.1-11.4)	9.2 (7.0-11.4)	8.9 (6.7-11.4)
CRP (mg/L)	29 (12-70)	38 (11-77)	33 (11-72)
Asbestos Exposed Controls			
Number	n=110	n=83	n=95
Age	71 (67-77)	71 (67-75)	72 (67 - 77)
Male Sex	103/110 (94%)	77/83 (93%)	90/95 (95%)
Known Asbestos Exposure	110/110 (100%)	83/83 (100%)	95/95 (100%)

NOTE: Values reported are median (interquartile range) unless otherwise stated. The eGFR, WCC, and CRP measurements and pleural fluid aspiration were not performed in asbestos-exposed controls.

CRP: C-reactive protein; eGFR: estimated glomerular filtration rate; SPM, suspected pleural malignancy; WCC: white cell count.

a Stage was recorded in 147/152 (97%) MPM patients

b SPM: Suspected Pleural Malignancy

### Test Results

#### SOMAscan

**Figure 2:**
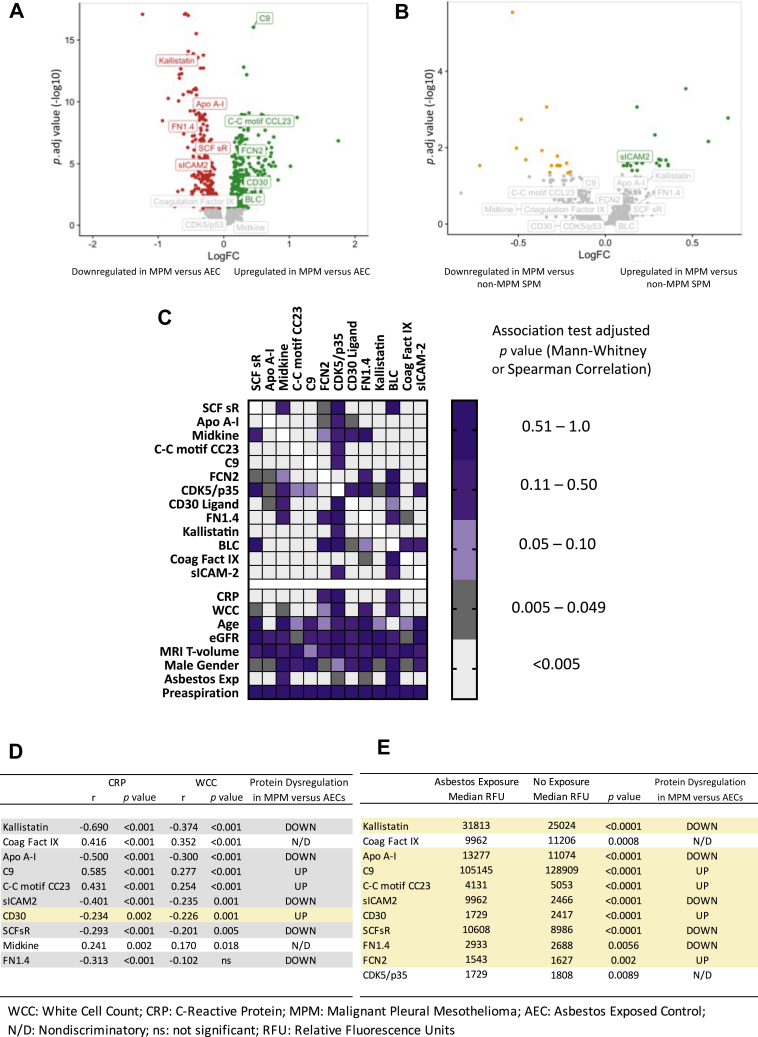
The SOMAscan proteomic assay was used to measure 1305 proteins including the 13 proteins in the signature previously reported by Ostroff et al.^10^*A* and *B* summarize differential protein expression (Limma [version 3.46] R package) between *(A)* MPM versus AECs and *(B)* MPM versus non-MPM pleural disease. Colored dots signify fold changes exceeding the FDR (*p.* adj. < 0.05). In Panel A (MPM versus AEC), all four of the proteins down-regulated in the original 13-protein classifier were also down-regulated here (APO A-1), FN1.4, mast/SCFsr (Kallistatin). In addition, four of the nine originally up-regulated proteins were up-regulated (C9, C-C motif CC23, FCN2, CD30 ligand). One protein (sICAM-2) that was originally up-regulated in MPM was down-regulated here. *B* reflects that the differential expression of the 13-protein signature was nondiscriminatory in MPM versus non-MPM pleural disease, with all but one protein falling below the FDR. *C* is a heatmap of adjusted *p* values summarizing associations observed among the 13 constituent SOMAscan proteins and associations with primary tumor volume (derived by MRI), age, eGFR, CRP, WCC, known asbestos exposure, and the timing of blood sampling (prefluid or postfluid aspiration). Notable associations were observed among constituent proteins and between proteins and CRP, WCC, and asbestos exposure, which correlated with 10, 9, and 11 of 13 proteins, respectively. Age, sex, eGFR, and the timing of blood sampling were generally not associated with protein levels. *D* and *E* summarize the degree of concordance between inflammatory (*D*) and asbestos Exp (*E*) relationships and the pattern of protein dysregulation in MPM versus AEC cases. Concordant relationships are highlighted in gray rows, discordant relationships in yellow rows. In *D*, 9 of 10 inflammatory correlations were concordant with protein dysregulation; that is, protein intensities were higher in cases with higher CRP and WCC and vice versa, with the exception being CD30 ligand. In *E*, all 11 asbestos Exp associations were discordant with the pattern of protein dysregulation—that is, the median value of signature proteins up-regulated in the MPM versus AEC signature tended to be lower in cases reporting asbestos Exp, and vice versa. It should be noted that the “asbestos exposure” group comprised of any SPM case that reported asbestos Exp (regardless of whether they were subsequently diagnosed with MPM, non-MPM malignancy, or any benign pleural disease), plus all AECs. These data implicate systemic inflammatory processes, but not asbestos Exp nor MPM primary tumor volume as important determinants of the proteomic differences observed in MPM cases compared with AECs. AEC, asbestos-exposed control; APO, apolipoprotein; C9, complement component 9; C-C motif CC23, C-C motif chemokine 23; CRP, C-reactive protein; eGFR, estimated glomerular filtration rate; Exp, exposure; FCN2, ficolin-2; FDR, false discovery rate; FN1.4, fibronectin fragment 4; MPM, malignant pleural mesothelioma; MRI, magnetic resonance imaging; N/D, nondiscriminatory; *p*. adj., adjusted *p*; RFU, relative fluorescent unit; SCFsr, stem cell growth factor receptor Kit; SPM, suspected pleural malignancy; WCC, white cell count.

Assay outputs for all 1305 proteins and associated data are available through the corresponding author. The outputs for the 13 proteins in the signature are reported in the appendix (Supplementary Appendix Table A1). Differential expression across all 1305 proteins between MPM versus AECs and MPM versus non-MPM SPM is summarized by volcano plots (Fig. 2*A* and *B*, respectively). In MPM versus AECs, all four of the proteins down-regulated in the original 13-protein classifier were down-regulated here. In addition, four of the nine originally up-regulated proteins were up-regulated, whereas one protein that was originally up-regulated in MPM was down-regulated here (Fig. 2*A* for detailed description). The 13-protein signature was associated with a training set AUC of 0.955 (0.926–0.984), 87.4% sensitivity, 92.4% specificity, and a validation set AUC of 0.855 (0.741–0.970), 75% sensitivity, 88.2% specificity (Supplementary Appendix Fig. A.3 for associated ROC curves). Differential protein expression comparing MPM and non-MPM SPM cases was nondiscriminatory (Fig. 2*B*); therefore, a random forest model was not generated. MPM histologic subtype was not associated with protein expression (Supplementary Appendix Fig. A4). Additional discovery analyses reporting expression differences between MPM and specific other diagnoses, including benign asbestos pleurisy will be reported separately.

#### Fibulin-3

**Figure 3:**
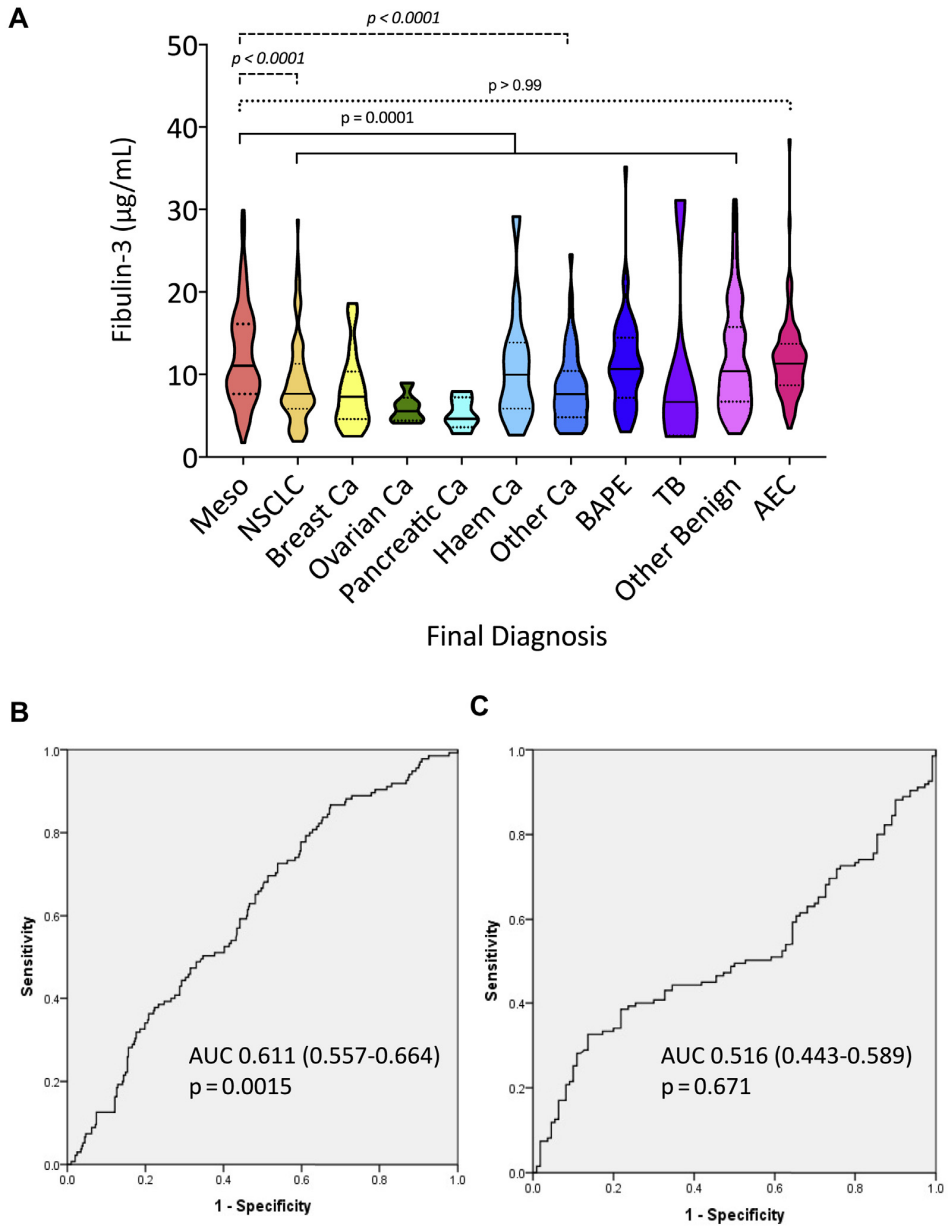
Plasma fibulin-3 was measured by ELISA (BosterBio, Pleasanton, CA) before diagnostic sampling in patients with SPM and a cohort of AECs, generating results in 135 patients with MPM, 403 patients with the non-MPM pleural disease, and 110 AECs. *A* reflects violin plots summarizing between-group differences, measured by Kruskal-Wallis test, with Dunn’s test for multiple comparisons. Fibulin-3 levels were significantly higher in MPM (11.06 μg/mL [7.63–16.12]) than in non-MPM SPM cases (8.96 μg/mL [6.01–13.66], *p* = 0.0001, see solid line bracket) but were not significantly different in AECs (11.31 μg/mL [8.68–13.72], *p* > 0.99, see dotted line bracket). The difference in the amalgamated non-MPM patient group reflected lower values in only the NSCLC (7.66 μg/mL [5.83–11.28], *p* < 0.0001) and “Other Cancer” groups (7.67 μg/mL [4.86–10.45], *p* < 0.001), see dashed line bracket. The latter comprised 61 cases with 13 different cancers, of which 14 of 61 (23%) were SCLC. *B* and *C* reveal ROC curves exhibiting AUC values of 0.611 ([0.557–0.664], *p* = 0.0015) and 0.516 ([0.443–0.589], *p* = 0.671) in differentiating MPM from non-MPM SPM and AEC, respectively. AEC, asbestos-exposed control; AUC, area under the curve; BAPE, benign asbestos pleural effusion; Ca, cancer; Meso, mesothelioma; MPM, malignant pleural mesothelioma; ROC, receiver operator characteristic; SPM, suspected pleural malignancy; TB, tuberculous pleuritis.

Fibulin-3 levels were significantly higher in MPM (11.06 μg/mL [7.63–16.12]) than in non-MPM SPM cases (8.96 μg/mL [6.01–13.66], *p* = 0.0001) but were not significantly different in AECs (11.31 μg/mL [8.68–13.72]) *p* > 0.99, which includes subgroups) (Fig. 3*A*). Per protocol, validation of the previous CloudClone ELISA cut-point (52 ng/mL)^3^ was not possible. Sensitivity at the prespecified 95% specificity level was 7.4% (cut-point >21.9 μg/mL, AUC = 0.611 [0.557–0.664], *p* = 0.0015) (Fig. 3*B*) in MPM versus non-MPM SPM and 11.9% (cut-point >20.11 μg/mL, AUC = 0.516 [0.443–0.589], *p* = 0.671) (Fig. 3*C*) in MPM versus AECs. Optimal sensitivity and specificity by Youden’s Index^22^ were 65.2% and 51.9% in MPM versus non-SPM (cut-point >9.12 μg/mL) and 40.0% and 74.5% in MPM versus AECs (cut-point >13.55 μg/mL). These results were robust in sensitivity analyses regarding handling of evolvers (Supplementary Appendix Fig. A5). Histologic subtype did not meaningfully affect assay performance. These data are presented in detail in the online Supplementary Data (Supplementary Appendix Fig. A6).

#### Mesothelin

**Figure 4:**
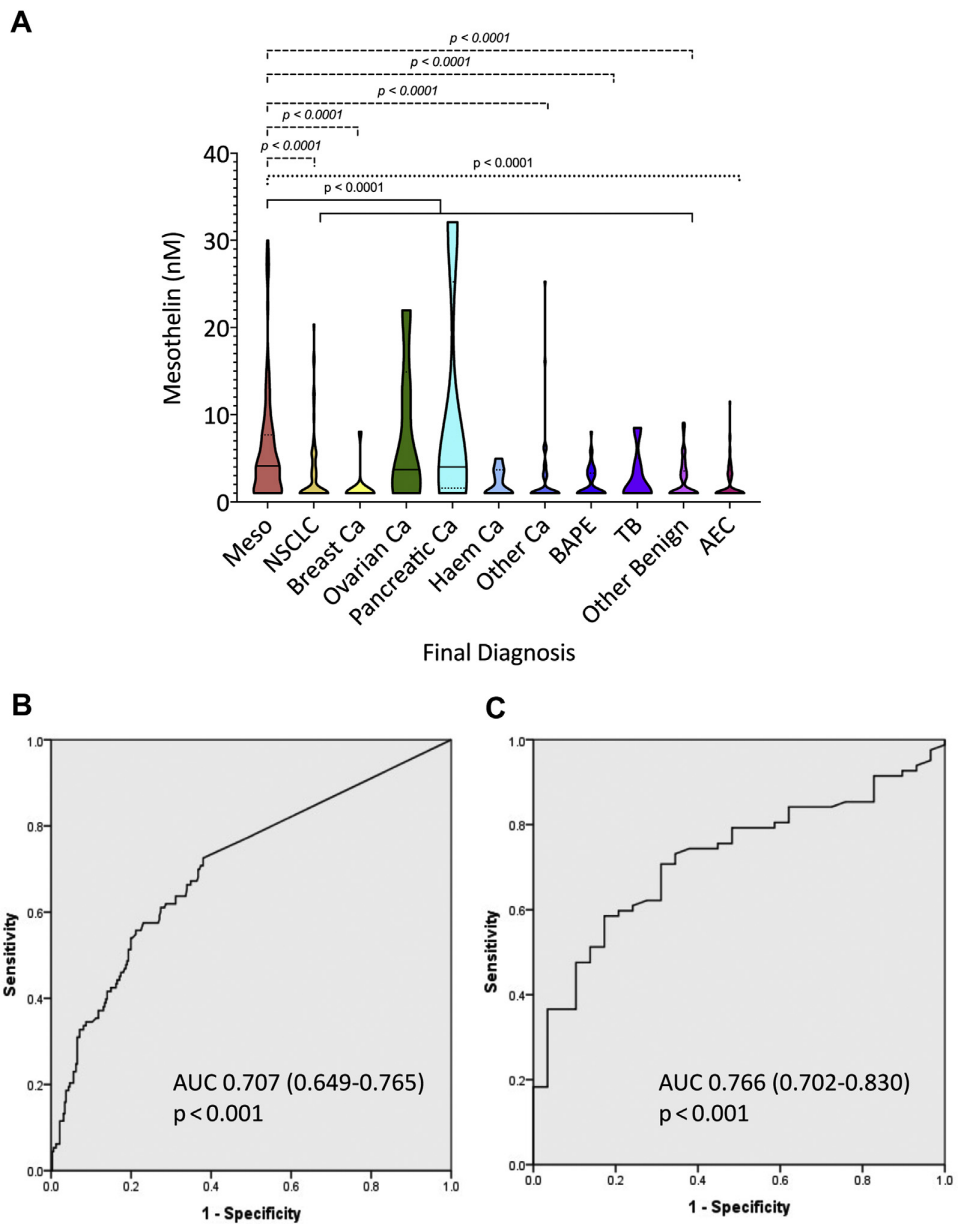
Serum mesothelin was measured by Mesomark ELISA (Fujirebio Diagnostics, Malvern, PA) before diagnostic sampling in patients with SPM and a cohort of AECs, generating results in 113 patients with MPM, 321 patients with the non-MPM pleural disease, and 95 AECs. *A* exhibit violin plots summarizing between-group differences, measured by Kruskal-Wallis test, with Dunn’s test for multiple comparisons. Mesothelin levels were significantly higher in MPM cases (4.12 nM [1.0–7.69]) than in non-MPM SPM (1.0 nM [1.0–3.52], see solid line bracket) and AECs (1.0 nM [1.0–3.01], both *p* < 0.0001, see dotted line bracket). The difference in the amalgamated non-MPM patient group reflected lower values in NSCLC, breast cancer, other cancers, BAPE, and “other benign” groups (all *p* < 0.0001 as highlighted by dashed line brackets). *B* and *C* reveal ROC curves exhibiting AUC values of AUC 0.707 ([0.649–0.765], *p* < 0.001) and 0.766 ([0.702–0.830], *p* < 0.001) in differentiating MPM from non-MPM SPM and AECs, respectively. AEC, asbestos-exposed control; AUC, area under the curve; BAPE, benign asbestos pleural effusion; Ca, cancer; Meso, mesothelin; MPM, Malignant pleural mesothelioma; ROC, receiver operator characteristic; SPM, suspected pleural malignancy; TB, tuberculous pleuritis.

Mesothelin levels were significantly higher in MPM (4.12 nM [1.0–7.69]) than in non-MPM SPM (1.0 nM [1.0–3.52]) and AECs (1.0 nM [1.0–3.01], both *p* < 0.0001, which includes subgroups) (Fig. 4*A*). These results were robust in planned sensitivity analyses evaluating handling of results below the assay’s LLOQ (Supplementary Appendix Table A.2) except when all less than LLOQ values (56%) were excluded, which was not used. Sensitivity and specificity at the prespecified 2.5 nM cut-point were 69.9% and 63.2% in MPM versus non-MPM SPM cases (AUC = 0.707 [0.649–0.765], *p* < 0.001) (Fig. 4*B*) and 69.9% and 70.5% in MPM versus AECs (0.766 [0.702–0.830], *p* < 0.001) (Fig. 4*C*). At the prespecified 95% specificity, sensitivity was 20.4% in MPM versus non-MPM SPM and 37.2% in MPM versus AECs. These results were robust in planned sensitivity analyses regarding the handling of evolvers (Supplementary Appendix Fig. A7). In sensitivity analyses regarding histologic subtype, sensitivity at 95% specificity was lower in nonepithelioid versus epithelioid MPM versus non-MPM SPM (11.9% versus 24.6%), but this was not clinically meaningful. These data are presented in detail in the online supplement (Supplementary Appendix Fig. A8).

### Relationship With MRI Tumor Volume

A total of 26 of 152 patients (17.1%) with MPM participated in the MRI substudy, exceeding the target sample size (n = 20). No statistically significant correlations between the primary tumor volume and any of the 13 constituent SOMAscan proteins (Fig. 2*C*), fibulin-3 (r = 0.0137, *p* = 0.502) or mesothelin (r = −0.031, *p* = 0.893) were observed.

### Effect of Potential Confounders

Notable relationships were identified among the 13 constituent SOMAscan proteins, and between proteins and CRP, WCC, and a history of asbestos exposure, in which the latter was recorded in all participants and is not synonymous with being an AEC. CRP, WCC, and asbestos exposure correlated with 10, 9, and 11 of 13 proteins, respectively (Fig. 2*C*). The direction of the inflammatory correlations was generally concordant with the pattern of protein dysregulation in MPM versus AEC; that is, signature protein intensities were higher in cases with higher CRP and WCC and vice versa, with the exception being CD30 ligand (Fig. 2*D*). The opposite was observed with asbestos exposure. Figure 2*E* reflects that the median value of signature proteins up-regulated in the MPM versus AEC signature tended to be lower in cases reporting asbestos exposure (and vice versa). In interpreting these data, it is important to note that in this analysis, the asbestos exposure group included any SPM with this history whatever their final diagnosis, plus the AEC group, all of whom reported this by definition.

Weak positive associations were observed between fibulin-3 and age (r = 0.189, *p* < 0.0001), male sex (median = 10.18 [6.9–14.59] μg/mL versus 8.66 [5.99–12.97] μg/mL in females, *p* = 0.006) and known asbestos exposure (median = 10.68 [7.56–14.86] μg/mL versus 8.04 [5.42–12.22] μg/mL, *p* < 0.0001). Fibulin-3 was negatively associated with eGFR (r = −0.251, *p* < 0.0001) and values were higher in blood drawn prepleural aspiration (median = 10.3 [6.61–15.36] μg/mL versus 8.94 [6.4–14.11] μg/mL, *p* = 0.023). There was no association between fibulin-3 and WCC (r = -0.087, *p* = 0.052) or CRP (r = 0.03, *p* = 0.519).

Mesothelin values were not associated with sex, asbestos exposure, or the timing of blood sampling, but were positively associated with age (r = 0.252, *p* < 0.0001) and negatively associated with eGFR (r = −0.307, *p* < 0.0001). There was a weak positive correlation between mesothelin and WCC (r = 0.117, *p* = 0.018) but no correlation with CRP (r = 0.055, *p* = 0.294).

### Evolvers

A total of 16 of 638 SPM cases (2.5%) were classified as evolvers. Four of 16, nine of 16, and 10 of 16 had SOMAscan, fibulin-3, and mesothelin values available, respectively. A total of three of the four SOMAscan results (75%) classified the patients as MPM. A total of six of nine fibulin-3 values (66.7%) were above the optimal MPM cut-point (>9.12 μg/mL). A total of seven of 10 mesothelin values (70%) were above the prespecified MPM cut-point (>2.5 nM), one of 10 was unclassifiable (mean value <3). The inclusion of evolver values did not affect the primary outcomes regarding fibulin-3 or mesothelin (Supplementary Appendix Figs. A3 and A4).

## Discussion

In this large, multicenter, prospective study, we recruited an intention-to-diagnose MPM population of 638 patients and 110 AECs from 22 centers in the United Kingdom and Ireland. Blood samples were collected before biopsy or treatment, according to a prespecified and standardized protocol^11^ that incorporated robust assay validation, diagnosis quality control, and mandatory 1-year follow-up of patients with benign biopsies. The study generated adequately powered, well-matched assay cohorts, facilitating a robust assessment of the SOMAscan proteomic assay and fibulin-3, with benchmarking against mesothelin.

In this setting, which has high internal and external validity, the SOMAscan proteomic assay performed well in differentiating MPM from AECs but was unable to differentiate patients with other pleural diseases (i.e., non-MPM SPM, which included non-MPM malignancy, and benign pleural disease). The evolution of the assay during recruitment precluded the planned per protocol validation of the version originally used by Ostroff et al.^9^ Nevertheless, the lack of any differential protein expression between patients with MPM and non-MPM effectively excludes any utility as a clinical diagnostic. The good performance observed in MPM versus AEC (75% sensitivity, 88.2% specificity, validation AUC = 0.855) supports a future role in screening, particularly given the emergence of effective MPM treatments that might soon justify such an approach.^23^^,^^24^ This is consistent with the original training of the SOMAscan model to discriminate MPM from AECs.^9^ More importantly, the protein dysregulation seen in MPM versus AECs was not driven by asbestos exposure per se, as detailed in Figure 2*E*, but similar analyses regarding CRP and WCC do implicate systemic inflammation as an important proteomic confounder (Fig. 2*D*). This is, perhaps, unsurprising given the inflammatory origins of MPM but predicts that the current signature’s moderate specificity (88.2%) may limit future screening applications because the positive predictive value will be lower in low prevalence settings and a lower cut-point may need to be used to increase sensitivity about the current 75%. Previous screening studies have tested various biomarkers, frequently mesothelin, but report low sensitivity (0%–20%), few MPM cases, and a high number of false-positive cases.^25^, ^26^, ^27^, ^28^ The AUC value for SOMAscan in MPM versus AEC reported here was higher than mesothelin (0.766), but future screening efforts may still need to mitigate against modest PPV, for example, by recruiting only persons at high MPM risk using models on the basis of occupational asbestos exposure,^29^^,^^30^ and interpreting results in light of inflammatory indices.

Our results reveal that fibulin-3 is not a useful diagnostic test for MPM. Sensitivity at the prespecified 95% specificity level was only 7.4% in differentiating MPM from non-MPM SPM (AUC = 0.611 [0.557–0.664, *p* = 0.0015) (Fig. 3). Discriminant performance was poorer in differentiating MPM and AECs and no better than chance in this context (AUC = 0.516 [0.443–0.589], *p* = 0.415). These data were inferior to mesothelin, which is not recommended for diagnostic use owing to low sensitivity.^7^ This guidance is supported by our data, which revealed that mesothelin sensitivity at 95% specificity was only 20.4% in MPM versus non-MPM SPM and 37.2% in MPM versus AECs.

Fibulin-3 results in our study were generated using the BosterBio ELISA, not the CloudClone assay used in previous reports.^2^^,^^3^^,^^5^^,^^6^ This was after a rigorous assessment of the CloudClone assay, which proved unacceptably inconsistent (Supplementary Appendix Fig. A2). This led to delay, required additional funding, and precluded the planned per protocol validation of the CloudClone assay and its associated cut-point (52 ng/mL).^2^ Nevertheless, our findings robustly reveal that fibulin-3 is not a useful diagnostic test, nor should it be used for screening asbestos-exposed individuals. Moreover, our experiences likely explain the previous conflicting data regarding fibulin-3 generated using the CloudClone ELISA. After the initial report of 96.7% sensitivity and 95.5% specificity,^2^ two small single-center studies reported similar outcomes (Agha et al.*,*^6^ sensitivity 100%, specificity 77.8%, n = 45 and Kaya et al.*,*^4^ sensitivity 93%, specificity 90%, n = 43). However, these were at high risk of selection bias, used serum rather than the plasma, and recruited as per convenience rather than intention-to-diagnose cohorts. In a larger two-center study, Creaney et al.^3^ reported lower performance (sensitivity = 22%, specificity = 95% at 52 ng/mL cut-point, n = 153), which was replicated using similar methods by Kirschner et al.^5^ (sensitivity = 13.5%, specificity = 96.9%, n = 69 [Sydney]; sensitivity = 12.7%, specificity = 87.5% n = 71 [Vienna]). These reports are more concordant with results from an external validation cohort originally reported by Pass et al.^2^ (sensitivity 33% at 100% specificity).^2^ Because the current study design controls for most sources of bias (see below), is adequately powered, and also addresses the analytical inconsistencies of the CloudClone ELISA it constitutes the most robust evaluation of fibulin-3 in MPM to date.

The current design minimized common sources of bias and imprecision^31^^,^^32^ and adhered to Standards for Reporting Diagnostic Accuracy guidelines^12^ throughout. Bias was minimized by recruiting an intention-to-diagnose MPM population, the use of broad eligibility criteria (reflected in the recruitment of 60.1% of those screened), and robust diagnostic assessment with minimal exclusions. Exclusions were only allowed when MPM evolved during 1-year follow-up and sensitivity analyses were robust when these were included (Supplementary Appendix Figs. A3 and A4). The incidence of MPM reported here (24.4%) is higher than in many countries. Although lower prevalence would increase negative predictive values, the clearly insufficient diagnostic sensitivity of both markers could not plausibly offset this. The poor diagnostic performance reported is consistent with the lack of any correlation with primary tumor volume. The MRI technique used to measure this is indirect but robust and outperforms both clinical stage and computed tomography volumetry as a prognostic indicator in MPM.^18^

In conclusion, in this study, which, to our knowledge, is the largest prospective MPM biomarker study to date, the SOMAscan proteomic assay proved useful as a potential future screening test for MPM in asbestos-exposed persons. Neither fibulin-3 nor SOMAscan proved useful in the diagnosis of MPM.
