# Supplementary material for: Serum Proteomics and Plasma Fibulin-3 in Differentiation of Mesothelioma From Asbestos-Exposed Controls and Patients With Other Pleural Diseases
Source: J Thorac Oncol. 2021 Oct;16(10):1705–17. doi: 10.1016/j.jtho.2021.05.018 (PMC8514249; doi:10.1016/j.jtho.2021.05.018)
Supplement: Supplementary Figures 1-8, and Supplementary Tables 1 and 2 [file mmc1.docx]

**SERUM PROTEOMICS AND PLASMA FIBULIN-3 IN DIFFERENTIATION OF MESOTHELIOMA FROM ASBESTOS-EXPOSED CONTROLS AND PATIENTS WITH OTHER PLEURAL DISEASES**

**ONLINE ONLY SUPPLEMENT: APPENDIX 1**

**Contents**

Figure A.1 Study Flowchart Page 1

Figure A.2 Laboratory Validation of Fibulin-3 Assays Page 2

Figure A.3 SOMAscan training and validation set Page 3

ROC curves

Figure A.4 SOMAscan association with histological Page 4

sub-type

Figure A.5 Fibulin-3: Evolver Sensitivity Analyses Page 5

Figure A.6 Fibulin 3: Histological subtype Sensitivity Page 6

Analyses

Figure A.7 Mesothelin: Evolver Sensitivity Analyses Page 7

Figure A.8 Mesothelin: Histological subtype Sensitivity Page 8

Analyses

Table A.1 SOMAscan assay outputs Page 9-13

Table A.2 Mesothelin: <LLOQ Sensitivity Analyses Page 14

**Figure A.1**

Summary of the design of the DIAPHRAGM study. Panel A describes the optimal diagnostic pathway for patients presenting with pleural effusion +/- pleural thickening or a pleural mass. Panel B describes the optimal diagnostic pathway for patients who present with an isolated pleural mass, but no significant fluid component. The MRI sub-study was only available to patients presenting with pleural effusion to centers in the West of Scotland.

**Figure A.2**

**Panels A** and **B** report intra-assay reproducibility for the CloudClone Fibulin 3 ELISA (Panel A) and the BosterBio Fibulin 3 ELISA (Panel B). Standard curves were generated on 5 separate occasions (each represented by a different solid shape) with each concentration of standard tested in duplicate, according to the manufacturer’s instructions. Each individual standard curve was analysed by linear regression of the duplicate standards. The intra-assay reproducibility of the CloudClone assay (Panel A) was deemed unacceptable. The performance of the BosterBio assay (Panel B) was deemed acceptable.

**Panel C** summarises data regarding the inter-assay precision of the two assays. This is reported as coefficient of variation (CV%), based on measurement of low, medium and high-quality control (QC) standards on 5 separate occasions according to the manufacturers’ instructions. The precision of the CloudClone assay was deemed unacceptable, while the performance of the BosterBio assay was deemed acceptable.

**Panel D** summarises integrated data on precision and reproducibility for each assay using patient samples. Each sample (Study ID in the far-left column) was analysed in duplicate on 2 separate occasions (n=4), according to the manufacturers’ instructions for each assay with Fibulin-3 concentrations determined by extrapolation from standard curves. For the BosterBio assay, samples were diluted 1:1000 to fit within the standard curve range. Measurements using the CloudClone assay were performed in undiluted samples. The performance of the CloudClone assay was again deemed insufficient (N/D=not determined).

**Figure A.3**

The SOMAscan® proteomic assay was used to measure 1305 proteins, including the 13 proteins in the signature previously reported by Ostroff et al. A random forest model was constructed for MPM v non-MPM SPM cases, which were split 80:20 into training and internal validation sets. Five repeats of 10-fold cross-validation were used for training. Model performance was evaluated in a gradient boosted logistic regression model and reported as Area Under the Curve (AUC (95% DeLong confidence interval) and optimal sensitivity and specificity. **Panel A** shows the Receiver Operator Characteristics (ROC) curve for the training set (AUC 0.955 (0.926-0.984), 87.4% sensitivity, 92.4% specificity). **Panel B** shows the ROC curve for the validation set (AUC 0.855 (0.741-0.970), 75% sensitivity, 88.2% specificity).

**Figure A.4**

The SOMAscan® proteomic assay was used to measure 1305 proteins, including the 13 proteins in the signature previously reported by Ostroff et al. No association was found between expression of any of the 13 proteins and histological subtype in 119 patients with MPM (Epithelioid n=68, Sarcomatoid n=14, Biphasic n=15, NOS/Not recorded n=22). The heatmap depicts expression of each protein (y axis) for each meso patient (x axis). Values were centre scaled per-protein then trimmed using 1.5 x IQR method. Values above the mean are represented in red and below in blue. There is no group of proteins where one subtype shows remarkable pattern of increased/decreased levels of any of the proteins. However, the 13-protein signature on the y axis can be split into two clades, the top clade including Apo A-I, Kallistatin, sICAM2 & SCF sR and the bottom clade containing the rest (and no simplicifolious or outlier proteins). Additionally, hierarchical clustering within subtypes indicates that there is a more striking pattern where each subtype appears to have two primary groups of patients who have overexpression of one protein clade or the other, and therefore have a similar pattern of protein levels.

**
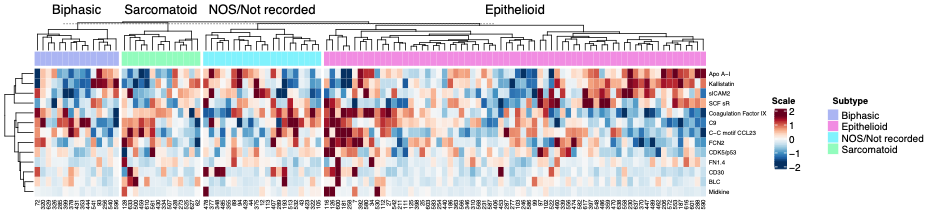
**

**Figure A.5**

Plasma Fibulin-3 was measured by ELISA (BosterBio, California, USA) prior to diagnostic sampling in patients with suspected pleural malignancy (SPM) and a cohort of asbestos exposed controls (AECs), generating results in 135 patients with Malignant Pleural Mesothelioma (MPM), 403 patients with non-MPM pleural disease and 110 AECs. 9 cases initially diagnosed with benign pleural disease who subsequently developed MPM within 12-months were defined as ‘evolvers’. Sensitivity analyses were performed to determine the effect on Receiver Operator Characteristic (ROC) performance when these cases were **(1) excluded as reported in the primary analysis**, **(2) included and classified as MPM** and **(3) included and classified as non-MPM SPM**. ROC performance of the disease classifications reported was not significantly different in these analyses.

**Figure A.6**

Plasma Fibulin-3 was measured by ELISA (BosterBio, California, USA) prior to diagnostic sampling in patients with suspected pleural malignancy (SPM) and a cohort of asbestos exposed controls (AECs), generating results in 135 patients with Malignant Pleural Mesothelioma (MPM), 403 patients with non-MPM pleural disease and 110 AECs. Sensitivity analyses were performed to determine the effect of MPM histological subtype on Receiver Operator Characteristic (ROC) performance. 75/135 (56%) MPM cases were epithelioid, 51/135 (38%) were non-epithelioid and subtype was not specified in 9/135 (6%). There was no clinically meaningful difference in ROC performance in either subtype.

**Figure A.7**

Serum mesothelin was measured by Mesomark® ELISA (Fujirebio Diagnostics, Pennsylvania, USA) prior to diagnostic sampling in patients with suspected pleural malignancy (SPM) and a cohort of asbestos exposed controls (AECs), generating results in 113 patients with Malignant Pleural Mesothelioma (MPM), 321 patients with non-MPM pleural disease and 95 AECs. 10 cases initially diagnosed with benign pleural disease who subsequently developed MPM within 12-months were defined as ‘evolvers’. Sensitivity analyses were performed to determine the effect on Receiver Operator Characteristic (ROC) performance when these cases were **(1) excluded as reported in the primary analysis**, **(2) included and classified as MPM** and **(3) included and classified as non-MPM SPM.** ROC performance of the disease classifications reported was not significantly different in these analyses.

**Figure A.8**

Serum mesothelin was measured by Mesomark® ELISA (Fujirebio Diagnostics, Pennsylvania, USA) prior to diagnostic sampling in patients with suspected pleural malignancy (SPM) and a cohort of asbestos exposed controls (AECs), generating results in 113 patients with Malignant Pleural Mesothelioma (MPM), 321 patients with non-MPM pleural disease and 95 AECs. Sensitivity analyses were performed to determine the effect of MPM histological subtype on Receiver Operator Characteristic (ROC) performance. 61/113 (54%) MPM cases were epithelioid, 42/113 (37%) were non-epithelioid and subtype was not specified in 10/113 (9%). Sensitivity at 95% specificity was lower in non-epithelioid v epithelioid cases (11.9% v 24.6%) but this was not clinically meaningful.

**Table A.1**

The SOMAscan® proteomic assay utilises modified DNA aptamers, termed SOMAmers®, to bind 1305 proteins within 65µL of serum, using a bead-based microarray (version 3). This generates an output in relative fluorescent units (RFU), which is directly proportional to the amount of target protein. Assay outputs for the 13 constituent proteins in the MPM signature previously reported by Ostroff *et al* are shown below. Data for all 1305 proteins, and associated data are available on application to the PREDICT-Meso Network, via the corresponding author.

**Table A.2**

Outcome of Sensitivity Analyses performed to determine the effect on disease sub-group comparisons of different methods of handling values below the Lower Limit of Quantification (LLOQ) of the Mesothelin assay. Values reported are adjusted p-values for comparisons relative to the Malignant Pleural Mesothelioma (MPM) group, generated by Kruskal-Wallis tests, with Dunn's test for multiple comparisons. The total number of values available by each method are reported in the top right corner of each panel.

| Round to half (replace <'values' with half LLOQ) | | | |  | Round up (replace <'value' with value) | | | |
| --- | --- | --- | --- | --- | --- | --- | --- | --- |
| (Reported) | NB: Half LLOQ=1.0nM | | n=529 |  |  | |  | n=529 |
|  |  |  |  |  |  | |  |  |
| MPM vs. NSCLC | | <0.0001 |  |  | MPM vs. NSCLC | <0.0001 | | |
| MPM vs. Breast Ca | | <0.0001 |  |  | MPM vs. Breast Ca | <0.0001 | | |
| MPM vs. Ovarian Ca | | >0.9999 |  |  | MPM vs. Ovarian Ca | >0.9999 | | |
| MPM vs. Pancreatic Ca | | >0.9999 |  |  | MPM vs. Pancreatic Ca | >0.9999 | | |
| MPM vs. Haem Ca | | 0.2444 |  |  | MPM vs. Haem Ca | 0.2732 | | |
| MPM vs. Other Ca | | <0.0001 |  |  | MPM vs. Other Ca | <0.0001 | | |
| MPM vs. BAPE | | <0.0001 |  |  | MPM vs. BAPE | <0.0001 | | |
| MPM vs. TB |  | 0.5438 |  |  | MPM vs. TB | |  | 0.3387 |
| MPM vs. Other Benign | | <0.0001 |  |  | MPM vs. Other Benign | <0.0001 | | |
| MPM vs. AEC | | <0.0001 |  |  | MPM vs. AEC | <0.0001 | | |
|  |  |  |  |  |  | |  |  |
| Round down (replace <'value' with 0) | | |  |  | Exclude (exclude all cases with < values) | | | |
|  |  |  | n=529 |  |  | |  | n=233 |
|  |  |  |  |  |  | |  |  |
| MPM vs. NSCLC | | <0.0001 |  |  | MPM vs. NSCLC | >0.9999 | | |
| MPM vs. Breast Ca | | <0.0001 |  |  | MPM vs. Breast Ca | >0.9999 | | |
| MPM vs. Ovarian Ca | | >0.9999 |  |  | MPM vs. Ovarian Ca | >0.9999 | | |
| MPM vs. Pancreatic Ca | | >0.9999 |  |  | MPM vs. Pancreatic Ca | >0.9999 | | |
| MPM vs. Haem Ca | | 0.2444 |  |  | MPM vs. Haem Ca | 0.7137 | | |
| MPM vs. Other Ca | | <0.0001 |  |  | MPM vs. Other Ca | >0.9999 | | |
| MPM vs. BAPE | | <0.0001 |  |  | MPM vs. BAPE | 0.0276 | | |
| MPM vs. TB |  | 0.5438 |  |  | MPM vs. TB | |  | >0.9999 |
| MPM vs. Other Benign | | <0.0001 |  |  | MPM vs. Other Benign | 0.0246 | | |
| MPM vs. AEC | | <0.0001 |  |  | MPM vs. AEC | 0.0032 | | |
|  |  |  |  |  |  | |  |  |

NSCLC: Non-small Cell Lung Cancer; Ca: Cancer; BAPE: Benign Asbestos Pleural Effusion; TB: Tuberculous Pleuritis; AEC: Asbestos Exposed Control
